# Genetic evidence of dietary patterns and diet-derived antioxidants on polyp development through gut microbiota modulation

**DOI:** 10.1097/MD.0000000000045434

**Published:** 2025-10-31

**Authors:** Ying Zou, Cheng Zhou, Kaiting Sun, Shenkang Tang, Shuhan Yang, Guankai Lin

**Affiliations:** a Department of Gastroenterology, Changzhou Hospital of Traditional Chinese Medicine Affiliated to Nanjing University of Chinese Medicine, Changzhou, PR China; b Department of Oncology, Wenzhou Hospital of Integrated Traditional Chinese and Western Medicine, Wenzhou, PR China; c Department of Oncology, The First College of Clinical Medicine, Shaanxi University of Chinese Medicine, Xianyang, PR China.

**Keywords:** colorectal polyp, diet, female genital tract polyp, gastric polyps, Mendelian randomization, nasal polyp

## Abstract

Previous studies have shown that dietary habits are associated with the development of several diseases in humans. However, whether these factors contribute to polyps remains unknown. In this study, we employ Mendelian randomization (MR) to investigate the causal relationship between genetically predicted dietary factors, including diet-derived antioxidant intake, and 4 types of polyps: nasal polyps, female genital tract polyps, colorectal polyps, and gastric polyps, as well as to explore the mediating role of the gut microbiota. We identified genetic variants associated with 7 dietary sources of circulating antioxidants and 15 dietary intake-related factors using genome-wide association study data from the UK Biobank. Genome-wide association study data for the 4 polyp types were obtained from the FinnGen consortium. Two-sample MR analyses were conducted to identify dietary factors causally associated with polyps, and sensitivity analyses were performed to assess heterogeneity and horizontal pleiotropy. Finally, 2-step MR was used to mine potential intermediary gut microbiota. Significant causal associations were identified between specific dietary components/gut microbiota and polyp risk. A total of 4 causal pathways were identified in which dietary factors modulated gut microbiota and thus polyps. Increased intake of lobster/crab inhibited the proliferation of *Holdemania*, thereby increasing the risk of nasal polyps. Bread intake, a protective factor for colon polyps, acted by increasing the abundance of *Flavonifractor*. The intake of bacon inhibits the protective factor *Alcaligenaceae* in gastric polyps. Coffee intake increased the risk of female genital tract polyps by increasing the abundance of *Victivallaceae*. Our findings provide evidence of a causal effect of dietary intake and diet-derived antioxidants on polyps in humans, emphasizing the importance of gut microbiota during polypogenesis. Approaches that target these factors may offer bright prospects for the treatment and prevention of polyps.

## 1. Introduction

Polyps are abnormal tissue growths on the surface of human tissues, which may not only affect the quality of life, but also have the risk of malignant transformation. Depending on the site of occurrence, they can be categorized into nasal polyps, cervical polyps, colorectal polyps, gastric polyps, and other types. Most polyps have no obvious clinical symptoms, but in a few cases, they may exist in combination with other diseases such as chronic sinusitis and *Helicobacter pylori* infection. Nasal polyps are inflammatory growths of the mucosa of the paranasal sinuses caused by chronic mucosal inflammation, usually originating in the middle nasal passage and sieve bone region, with a prevalence of 1% to 4% in adults.^[1]^ The female genital tract comprises the vagina, uterus, fallopian tubes, and ovaries. Polyps in the female genital tract are more common in the uterus and cervix, while vaginal polyps are less common, and tubal and ovarian polyps are relatively rare. Studies have shown that the presence of polyps in the female genital tract may be associated with an increased risk of infertility.^[2]^ Colorectal polyps are formed due to abnormal proliferation of the normal mucosal epithelium and can be classified into different histological types, such as adenomatous polyps, hyperplastic polyps, and inflammatory polyps. Most colorectal polyps are benign, and their occurrence is associated with various risk factors, including genetic factors, environmental exposures, and lifestyle factors.^[3]^ However, adenomatous polyps carry a risk of cancer and require close monitoring. Gastric polyps often arise from abnormal proliferation of epithelial cells during the repair and regeneration of the gastric mucosa after injury, and are closely associated with chronic gastritis, *Helicobacter pylori* infection, and bile reflux. While most gastric polyps are benign, the risk of cancer is relatively high when the diameter exceeds 2 cm or when atrophic gastritis is present.^[4]^

Diet plays a crucial role in meeting the human body’s basic physiological needs. Dietary habits tend to vary across different regions. Numerous studies^[5–7]^ have demonstrated a close relationship between dietary factors and the risk of colorectal polyps and gastrointestinal tumors. Moreover, adopting a reasonable dietary pattern is crucial for reducing the risk of gastrointestinal diseases. Leng et al^[8]^ discovered that diet might contribute to the formation of gallbladder polyps by influencing metabolic indicators like body mass index (BMI) and blood lipid levels. Patients with chronic rhinosinusitis and nasal polyps frequently experience vitamin D_3_ deficiency.^[9]^ Lower vitamin D_3_ levels are associated with larger polyp size. Therefore, appropriate vitamin D_3_ supplementation may aid in controlling the development of nasal polyps. Rock et al^[10]^ found a positive correlation between fiber intake and estrogen levels, whereas higher BMI may lead to reduced estrogen concentrations. Obese populations tend to have lower progesterone levels, and diets high in sugar, saturated fat, and animal protein also inhibit progesterone production.^[11,12]^ Consequently, adequate fiber intake and BMI management may aid in regulating estrogen and progesterone levels, potentially influencing the development of polyps in the female genital tract.

Although previous studies have clearly demonstrated significant associations between dietary patterns and disease occurrence, particularly digestive system diseases, the specific mechanisms underlying these relationships remain to be fully elucidated.^[13]^ The gut microbiota, as a critical bioactive factor, may play a central regulatory role in these associations. Gut microbes, as key bioactive factors, may centrally regulate the aforementioned association. Upon entering the intestine, food is catabolized by the gut microbial community. By secreting specific enzymes, gut microorganisms efficiently decompose complex carbohydrates (e.g., dietary fiber) that are indigestible in the upper gastrointestinal tract. The microbial fermentation of dietary fiber produces short-chain fatty acids (SCFAs), which are the primary energy source for colonic epithelial cells and regulate lipid and glucose metabolism.^[14]^ Furthermore, gut microbiota can synthesize essential vitamins, including vitamin K and B vitamins such as folate and B12.^[15]^ In terms of intestinal immune regulation, gut-associated lymphoid tissue serves as the core hub of intestinal immunity,^[16]^ and the microbial antigens within it can interact with dendritic cells, macrophages and other innate immune cells interact, thereby inducing regulatory T cells differentiation and secretion of secretory immunoglobulin A play a key role in maintaining intestinal immune tolerance and defending against pathogen invasion.^[17]^ It is important to emphasize that dietary patterns specifically regulate the composition and function of the gut microbiota, thereby influencing host health. A diet high in saturated fat can significantly increase the relative abundance of *Bilophila wadsworthia* by promoting the conjugation of taurine with host-derived bile acids. This process can lead to impaired intestinal barrier function and immune cell infiltration.^[18]^ Conversely, omega-3 polyunsaturated fatty acid (omega-3 PUFA) intake can increase the abundance of beneficial bacteria, such as *Bifidobacterium, Lactobacilli*, and *Akkermansia muciniphila*, which promotes SCFAs secretion and enhances gastrointestinal barrier integrity.^[19]^ Furthermore, excessive intake of animal-derived protein can enrich proteolytic bacteria, such as *Clostridium, Bacillus*, and *Staphylococcus*.^[20]^ In contrast, vegetarian populations exhibit a higher abundance of plant polysaccharide-fermenting bacteria, such as *Lachnospiraceae*. These bacteria can further regulate intestinal metabolism and immune function by fermenting plant polysaccharides into SCFAs, such as butyrate.^[21,22]^

Causal association studies of dietary patterns, gut microbiota and polyps have significant complexity, and traditional observational studies are susceptible to confounding factors. Even more importantly, these studies can identify associations but fail to clarify causal directions, especially when exposures such as diet and gut microbiota are affected by numerous external factors. Mendelian randomization (MR) is an analytical approach that leverages the association between a specific exposure and genetic polymorphism to evaluate the causal relationship between exposure and outcome. Employing genetic variation as an instrumental variable (IV) for exposure helps circumvent confounding factors and reverse causation present in traditional observational studies. Current MR studies have confirmed the association of several diseases with dietary patterns and antioxidant intake, including venous thromboembolism,^[23]^ neurodegenerative diseases,^[24]^ and others. In addition, the association of gut microbiota with diseases such as alcoholic liver disease^[25]^ and cirrhosis^[26]^ was also supported by MR analysis, showing that the method has broad applicability in exploring dietary factors, gut microbiota and disease associations. However, there is no current research that systematically explores the causal relationship between dietary patterns and polyps, and there is even less mechanistic analysis of the potential mediating effect of gut microbiota.

In this study, we employed MR analyses to evaluate the causal relationship between the intake of 15 dietary sources and 7 diet-derived antioxidants, and the development of nasal polyps, female genital tract polyps, colorectal polyps, and gastric polyps, and attempted to reveal the mediating role of gut microbiota. By elucidating the links among dietary patterns, gut microbiota, and polyp formation, preventive strategies against polyp occurrence can be more effectively optimized through dietary choices.

## 2. Materials and methods

### 2.1. Study design

**Figure 1.**
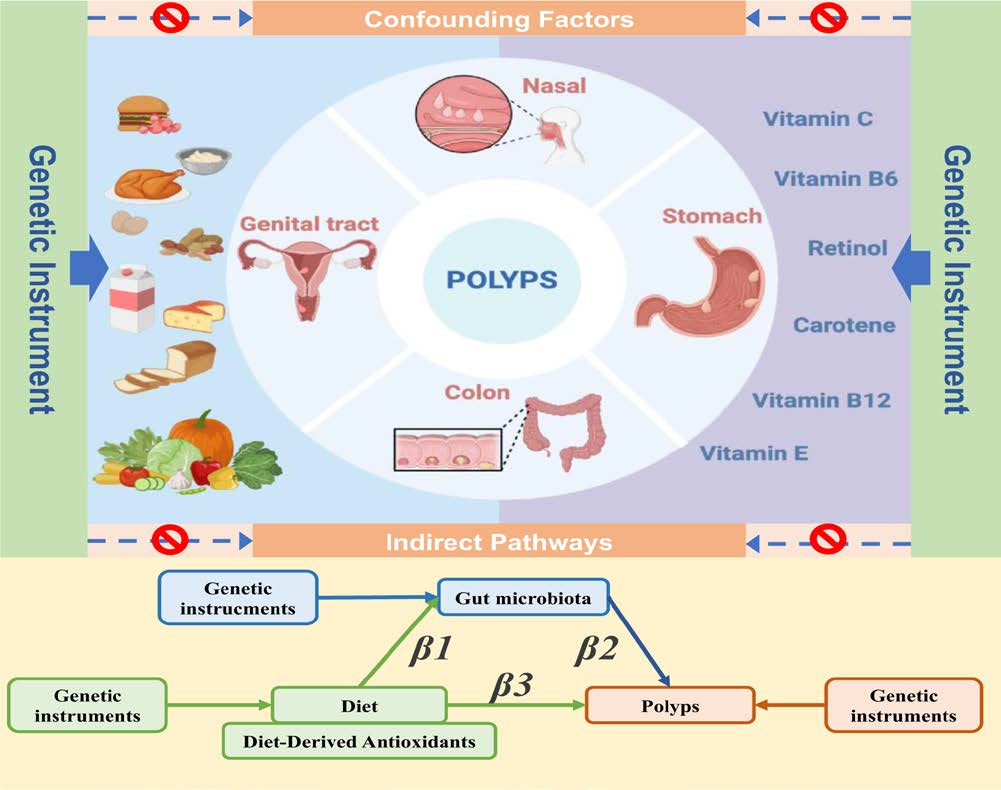
Overview of the design and methods of the study. Genetic instrumental variables were associated with only exposure factors and did not influence polyp outcomes independently of exposure factors and were not affected by confounding factors. Mediated Mendelian randomization analysis was used to assess the mediating role of gut microbiota in the causal relationship between dietary factors and polyps.

This study employed MR to investigate the association between 15 diets, 7 diet-derived antioxidants intake, and the development of 4 types of polyps in humans: nasal polyps, female genital tract polyps, colorectal polyps, and gastric polyps. Initially, we analyzed single nucleotide polymorphisms (SNPs) associated with dietary intake using univariate MR analyses, employing them as IVs. Furthermore, multinomial sensitivity analyses were conducted to enhance the robustness and reliability of the structural vector MR results. Finally, 2-step MR to determine the mediating effects of gut microbiota. The MR study was based on the following 3 assumptions^[27]^: SNPs are strongly associated with the exposure factors; SNPs are independent of potential confounding factors; and SNPs influence the outcome only through the exposure factors (Fig. 1).

### 2.2. Data sources

#### 2.2.1. GWAS summary statistics of exposure

We acquired diet-related exposure data from the IEU Open genome-wide association study (GWAS) program (https://gwas.mrcieu.ac.uk), which derived from the UK Biobank. This GWAS data pertains to dietary intake from over 5,00,000 individuals aged 40 to 69 years, collected by the UK Biobank between 2006 and 2010.^[28]^ The data for each dietary pattern comprised either integer variables (e.g., average number of cups of tea consumed per day) or categorical variables (e.g., frequency of beef consumption), with implausible responses being excluded.^[29]^ This study considered fifteen dietary factors, including milk intake, cereal intake, poultry intake, tea intake, fresh fruit intake, dried fruit intake, pork intake, bacon intake, cooked vegetable intake, lobster/crab intake, bread intake, coffee intake, cheese consumption, processed meat consumption, and beef intake, which collectively represent the dietary patterns of a significant portion of the global population. Additionally, 7 diet-derived antioxidants were included: carotene, retinol, vitamin B_6_, vitamin B_12_, vitamin C, vitamin D, and vitamin E. Detailed information regarding the diet-related exposure data is presented in Table 1. The GWAS data for gut microbiota were obtained from the MiBioGen consortium (https://mibiogen.gcc.rug.nl) and consisted of a total of 18,340 participants in 24 cohorts, including 13,266 in 16 European cohorts.^[30]^ The gut microbiota comprises a total of 211 taxa: 131 genus, 35 families, 20 orders, 16 classes and 9 phylums.

**Table 1:** Description of the contributing GWAS studies.

Traits	GWAS ID	Exposure	Sample size	*P*-value set	SNPs	Ancestry
Dietary patterns	ukb-b-2966	Milk intake	64,943	5.00E+06	21	European
	ukb-b-6066	Tea intake	4,47,485	5.00E+08	41	European
	ukb-b-5237	Coffee intake	4,28,860	5.00E+08	40	European
	ukb-b-15926	Cereal intake	4,41,640	5.00E+08	43	European
	ukb-b-11348	Bread intake	4,52,236	5.00E+08	32	European
	ukb-b-3433	Cake intake	64,949	5.00E+06	11	European
	ukb-b-3881	Fresh fruit intake	4,46,462	5.00E+08	55	European
	ukb-b-16576	Dried fruit intake	4,21,764	5.00E+08	43	European
	ukb-b-8089	Cooked vegetable intake	4,48,651	5.00E+08	17	European
	ukb-b-8006	Poultry intake	4,61,900	5.00E+08	8	European
	ukb-b-5640	Pork intake	4,60,162	5.00E+08	14	European
	ukb-b-2862	Beef intake	4,61,053	5.00E+08	17	European
	ukb-b-14746	Lobster/crab intake	64,938	5.00E+06	57	European
	ukb-b-4414	Bacon intake	64,949	5.00E+06	12	European
	ebi-a-GCST90096906	Cheese consumption	3,65,842	5.00E+08	17	European
	ebi-a-GCST90096921	Processed meat consumption	3,12,220	5.00E+08	6	European
Diet-derived antioxidants	ukb-b-16202	Carotene	64,979	5.00E+06	16	European
	ukb-b-17406	Retinol	62,991	5.00E+06	9	European
	ukb-b-7864	Vitamin B_6_	64,979	5.00E+06	18	European
	ukb-b-19524	Vitamin B_12_	64,979	5.00E+06	10	European
	ukb-b-19390	Vitamin C	64,979	5.00E+06	11	European
	ukb-b-18593	Vitamin D	64,979	5.00E+06	14	European
	ukb-b-6888	Vitamin E	64,979	5.00E+06	12	European

GWAS = genome-wide association study, SNPs = single nucleotide polymorphisms.

#### 2.2.2. GWAS summary statistics of outcome

Data for 4 polyps, including nasal polyps (6841 cases and 3,08,457 controls), female genital tract polyps (15,690 cases and 1,11,583 controls), colorectal polyps (22,254 cases and 3,89,927 controls), and gastric polyps (2901 cases and 4,09,280 controls), were obtained from the FinnGen Consortium^[31]^ (https://www.finngen.fi/fi). The FinnGen research program is a nationwide genetic study that collects a comprehensive range of genetic and electronic health record data, providing an important basis for investigating genetic variants associated with diseases.

### 2.3. Selection of genetic variants

The study involved diet-related 22 exposure factors, categorized into dietary patterns (15) and diet-derived antioxidants (7). For most dietary pattern exposures, GWAS data employed a stringent threshold of *P* < 5 × 10^−8^. However, milk intake, bacon intake, and lobster/crab intake had fewer than 5 SNPs meeting this threshold, necessitating the selection of SNPs with *P* < 5 × 10^−6^ as IVs. For diet-derived antioxidants, IVs were selected using SNPs with *P* < 5 × 10^−6^. An insufficient number of IVs could significantly reduce the statistical power of MR analysis and may lead to unstable estimates and bias. To ensure the feasibility of the analysis and obtain preliminary causal effect estimates, we had to adopt a slightly relaxed threshold. The sample sizes ranged from 62,991 to 4,62,630. Independent SNPs form the basis of MR studies. To exclude SNPs in linkage disequilibrium, a distance threshold of 10,000 kb and an *r*^2^ threshold of 0.001 were set. As in previous studies,^[26]^ we selected SNPs with *P* < 1 × 10^−5^ as IVs for gut microbiota. To exclude SNPs in chain imbalance, *k* = 500 kb and *r*^2^ = 0.1 were set. To mitigate the risk of bias from weak IVs, we calculated the *F*-statistic for each exposure factor. The *F*-statistics ranged from 14.59 to 646.73, indicating robust instrument strength (*F*-statistic > 10).^[32]^ Regarding potential sample overlap between exposure factors and outcomes, our literature review indicated no significant overlap between the GWAS data for exposures and outcomes.

### 2.4. Mendelian randomization analysis

We obtained summary data for dietary factors from the IEU Open GWAS, including SNP rsID, β value, effect allele, other allele, *P*-value, effect allele frequency, and sample size. The analyses were conducted using the TwoSampleMR,^[33]^ MR-PRESSO, and MendelianRandomization packages in R studio (version 4.3.2). For preliminary analyses, we employed the random-effects inverse variance weighted (IVW) method to estimate the potential effects of exposure factors on polyps, as it is the most widely used and robust analytical approach in MR studies. Additionally, MR-Egger, weighted median, simple mode, and weighted mode were employed for analyses.^[34–36]^ Heterogeneity tests were conducted to assess differences between IVs, and Cochran’s *Q* test was used to evaluate heterogeneity among the IVs. The MR-Egger intercept^[37]^ and MR-PRESSO methods^[38]^ were employed to assess horizontal pleiotropy. Furthermore, we confirmed the directionality of the MR using the mr_steiger2 function to validate the robustness of the genetic variance estimates.

In our study, we utilized a 2-step MR approach to explore the mediating influence of gut microbiota within the causal pathways linking dietary factors to the development of 4 distinct polyp types. Specifically, we dissected the overall impact of the exposure variable (dietary factors) on the outcome variable (polyps) into 2 components: a direct effect and an indirect effect mediated by the proposed mediator (gut microbiota). In the initial step, we identified genetic instruments associated with dietary factors and employed univariate MR to estimate their causal effects on candidate gut microbiota, denoted as β_1_. Subsequently, for the gut microbiota thus identified, we used independent genetic instruments to estimate their causal effects on polyp outcomes, referred to as β_2_. The total effect (β_3_) of each dietary factor on the respective polyp type was estimated through standard univariate MR analysis. The indirect effect mediated by gut microbiota was calculated as the product of the coefficients obtained from the 2 preceding steps, expressed as β indirect = β_1_*β_2_. The proportion of the total effect mediated by gut microbiota was then estimated as the ratio of the indirect effect to the total effect, β indirect/β_3_. A fundamental assumption underpinning this methodological framework is that the genetic instruments selected for the exposure variable do not influence the outcome variable through pathways independent of the hypothesized mediator (gut microbiota). To enhance the robustness of each causal estimate that forms the basis of our mediation analysis, we applied distinct MR methods across all univariate MR analyses. Additionally, we conducted comprehensive sensitivity analyses to further validate our findings. Furthermore, we employed the delta method to derive the standard errors and 95% confidence intervals (CIs) for the proportion mediated.

## 3. Results

### 3.1. Results of the weak instrumental variable test

The analyses included 22 exposure factors, comprising 15 dietary patterns and 7 diet-derived antioxidants. The number of SNPs ranged from 6 to 106, with the *F*-statistic for each SNP ranging from 20.87 to 646.73 (Supplementary file S1, Supplemental Digital Content, https://links.lww.com/MD/Q445). The *F*-statistics for each SNP in gut microbes ranged from 14.59 to 88.43, suggesting no potential for weak instrument bias (Supplementary file S2, Supplemental Digital Content, https://links.lww.com/MD/Q445).

### 3.2. Causal effects of dietary and diet-derived antioxidants intake on polyps

#### 3.2.1. Causal effects of dietary and diet-derived antioxidants on nasal polyps

**Figure 2.**
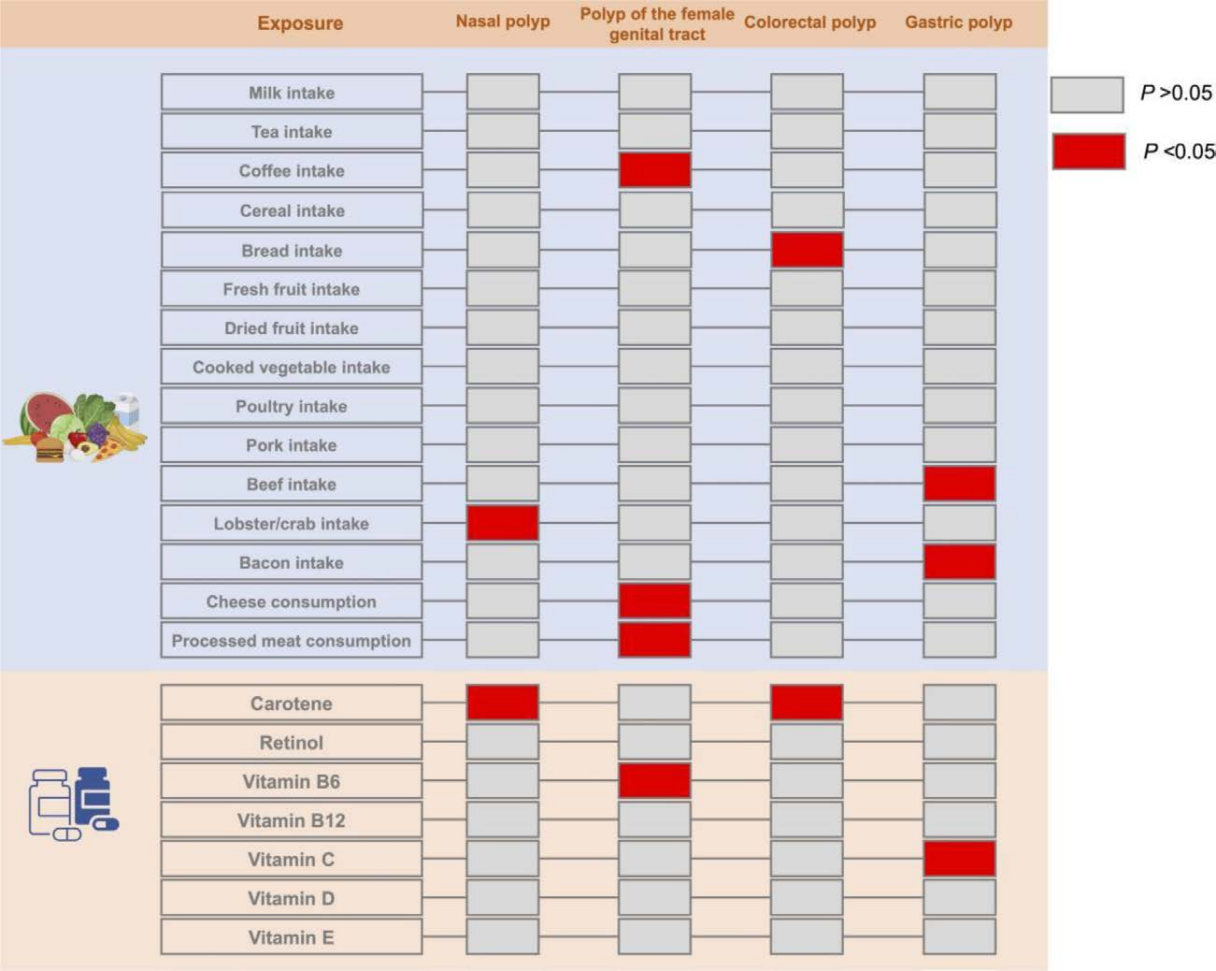
Causal relationship between dietary patterns and diet-derived antioxidants on 4 polyps. Red indicates *P* < .05 for IVW analysis in Mendelian randomization and gray indicates *P* > .05. *P* < .05 indicates that dietary patterns or diet-derived antioxidants have a causal relationship with polyps, while *P* > .05 suggests no causal relationship. IVW = inverse variance weighted.

**Figure 3.**
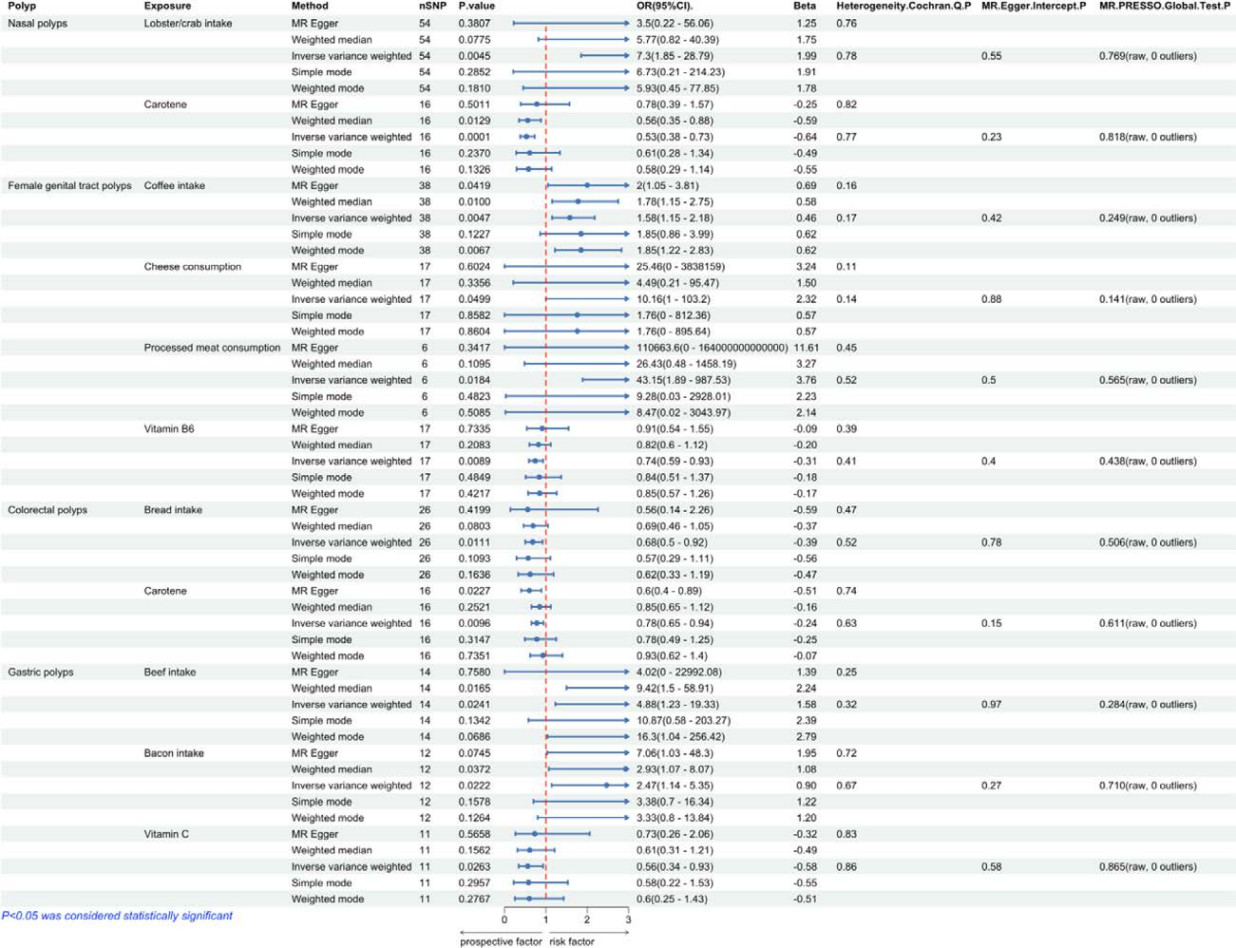
Univariate MR results between dietary factors and polyps. OR > 1 indicates increased risk, whereas < 1 indicates decreased risk. The last 3 columns are the analysis of horizontal pleiotropy and heterogeneity; *P* > .05 indicates no directed pleiotropy or heterogeneity. MR = Mendelian randomization, OR = odds ratio.

**Figure 4.**
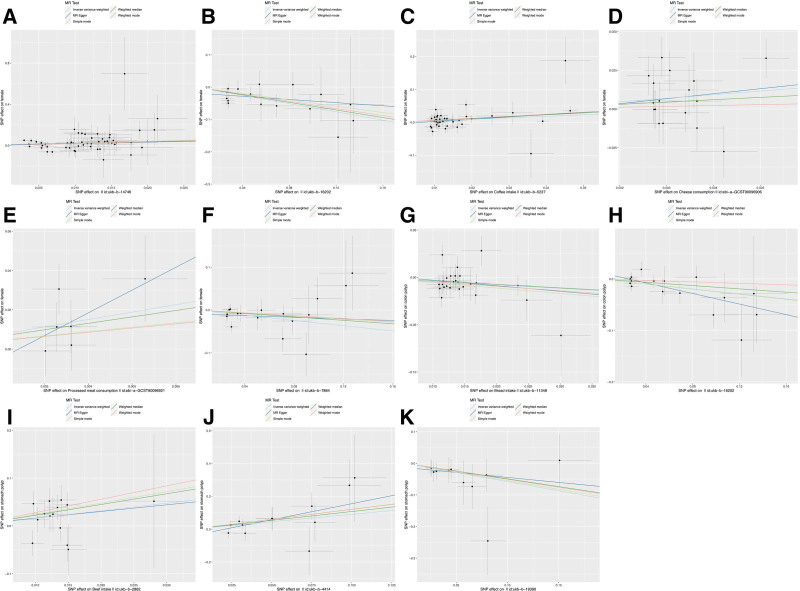
Scatter plots of the causal relationship between dietary factors and polyps. The *x*-axis represents the genetic association of each instrumental single nucleotide polymorphism (SNP) with the exposure (dietary patterns and diet-derived antioxidants), while the *y*-axis represents the genetic association of the same SNP with the outcome (polyps). Each data point on the plot corresponds to an individual SNP used as a genetic instrument. Five distinct MR analysis methods were fitted to these SNP data. All regression lines are constrained to pass through the origin, reflecting the fundamental MR assumption that the genetic instruments should not be associated with the outcome except through the exposure. The slope of each fitted line represents the causal effect estimate (β) for that particular method. The divergence in the slopes of these lines helps assess the robustness of the causal estimate and the potential influence of pleiotropic effects. (A) Lobster/crab intake and nasal polyps. (B) Carotene and nasal polyps. (C) Coffee intake and female genital tract polyps. (D) Cheese consumption and female genital tract polyps. (E) Processed meat consumption and female genital tract polyps. (F) Vitamin B_6_ and female genital tract polyps. (G) Bread intake and colorectal polyps. (H) Carotene and colorectal polyps. (I) Beef intake and gastric polyps. (J) Bacon intake and gastric polyps. (K) Vitamin C and gastric polyps. MR = Mendelian randomization, SNP = single nucleotide polymorphism.

MR analyses were conducted using 5 models: IVW, MR-Egger, simple median, weighted median, and weighted mode. Based on the IVW results, we identified 2 dietary factors causally associated with nasal polyp (Fig. 2). We found that lobster/crab intake (odds ratio [OR] = 7.30, 95% CI: 1.85–28.79, *P* = .0045) exhibited a potentially positive causal effect on nasal polyp, suggesting an increased risk of nasal polyps. In contrast, carotene (OR = 0.53, 95% CI: 0.38–0.73, *P* = .0001), a dietary source of antioxidants, may reduce the risk of nasal polyp (Fig. 3). Reverse MR analysis did not reveal a significant change in direction. The scatter plot of SNPs is shown in Figure 4.

#### 3.2.2. Causal effects of dietary and diet-derived antioxidants on female genital tract polyps

MR results showed that coffee intake (OR = 1.58, 95% CI: 1.15–2.18, *P* = .005), cheese consumption (OR = 10.16, 95% CI: 1.00–103.20, *P* = .0499), and processed meat consumption (OR = 4.31, 95% CI: 1.89–987.53, *P* = .0184) were risk factors for female genital tract polyps. Notably, despite the use of independent IVs selected at the stringent threshold of 5 × 10⁻⁸, sensitivity analyses revealed wide CIs for cheese consumption and processed meat consumption. In this context, the causal relationship may be unstable, and future research requires further stratification to narrow the CIs and determine the stability of these results. Among diet-derived antioxidants in vitamin B_6_ (OR = 0.74, 95% CI: 0.59–0.93, *P* = .0089) were found to be a protective factor for female genital tract polyps (Fig. 3). Reverse MR did not reveal significant directional changes. The scatter plot of SNPs is shown in Figure 4.

#### 3.2.3. Causal effects of dietary and diet-derived antioxidants on colorectal polyps

As shown in Figure 3, bread intake (OR = 0.68, 95% CI: 0.50–0.92, *P* = .0111) exhibited a protective effect against the development of colorectal polyps. Notably, carotene exhibited an inhibitory effect on both nasal polyps and colorectal polyps (OR = 0.53, 95% CI: 0.38–0.73, *P* = .0001). Inverse MR analysis did not reveal any significant findings. The scatter plot of SNPs is shown in Figure 4.

#### 3.2.4. Causal effects of dietary and diet-derived antioxidants on gastric polyps

A total of 2 diet habits were associated with an increase in gastric polyps, including beef intake (OR = 4.88, 95% CI: 1.23–19.33, *P* = .0241), bacon intake (OR = 2.47, 95% CI: 1.14–5.35, *P* = .0222). 1 diet-derived antioxidants reduced the risk of gastric polyps: vitamin C (OR = 0.56, 95% CI: 0.34–0.93, *P* = .0263; Fig. 3). Inverse MR analysis did not show significance. The scatter plot of SNPs is shown in Figure 4.

### 3.3. Causal effects of gut microbiota on polyps

#### 3.3.1. Causal effects of gut microbiota on nasal polyps

**Figure 5.**
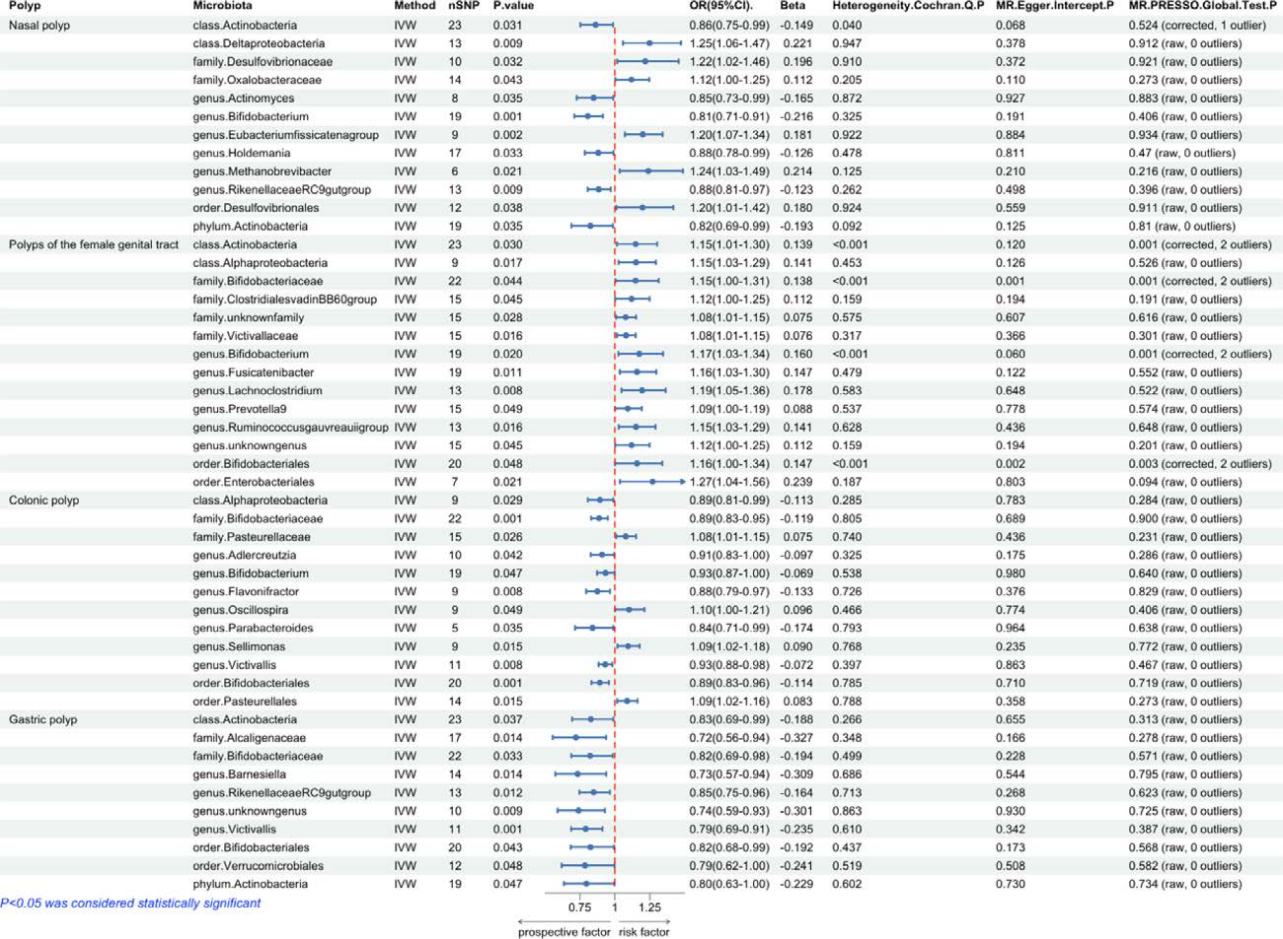
Univariate MR results between gut microbiota and polyps. OR > 1 indicates increased risk, whereas <1 indicates decreased risk. The last 3 columns are the analysis of horizontal pleiotropy and heterogeneity; *P* > .05 indicates no directed pleiotropy or heterogeneity. MR = Mendelian randomization, OR = odds ratio.

According to the IVW method we identified a total of 35 gut microbiota that were significantly causally associated with at least 1 type of polyp, including 12 microbial taxa that may be associated with nasal polyps (Fig. 5). In assessing the causal effect of gut microbiota on nasal polyps, we identified 1 phylum, 1 class, and 4 genus with an inhibitory effect on nasal polyp development, including phylum. *Actinobacteria* (OR = 0.82, 95% CI: 0.69–0.99, *P* = .035), genus.*Actinomyces* (OR = 0.85, 95% CI: 0.73–0.99, *P* = .035), genus.*Holdemania* (OR = 0.88, 95% CI: 0.78–0.99, *P* = .033), genus.*RikenellaceaeRC9gutgroup* (OR = 0.88, 95% CI: 0.81–0.97, *P* = .009), genus.*Bifidobacterium* (OR = 0.81, 95% CI: 0.71–0.91, *P* = .001). 1 class, 1 order, 2 families and 2 genus were risk factors for the development of nasal polyps, class.*Deltaproteobacteria* (OR = 1.25, 95% CI: 1.06–1.47, *P* = .009), order.*Desulfovibrionales* (OR = 1.2, 95% CI: 1.01–1.42, *P* = .038), family.*Oxalobacteraceae* (OR = 1.12, 95% CI: 1.00–1.25, *P* = .043), family.*Desulfovibrionaceae* (OR = 1.22, 95% CI: 1.02–1.46, *P* = .032), genus.*Methanobrevibacter* (OR = 1.24, 95% CI: 1.03–1.49, *P* = .021), genus.*Eubacteriumfissicatenagroup* (OR = 1.2, 95% CI: 1.07–1.34, *P* = .002). Inverse MR analysis did not show significance. Although the *P*-value for class.*Actinobacteria* (OR = 0.86, 95% CI: 0.75–0.99, *P* = .031) demonstrates statistical significance, its causal relationship is not supported due to the presence of heterogeneity. Moreover, after removing 1 outlier, the *P*-value becomes nonsignificant, further undermining the evidence for causality.

#### 3.3.2. Causal effects of gut microbiota on female genital tract polyps

According to the IVW method of univariate MR we identified a total of 14 gut microbiota that may be associated with female genital tract polyps, all of which are risk factors for polyp development (Fig. 5). class.*Alphaproteobacteria* (OR = 1.15, 95% CI: 1.03–1.29, *P* = .017), order.*Enterobacteriales* (OR = 1.27, 95% CI: 1.04–1.56, *P* = .021), family.*ClostridialesvadinBB60group* (OR = 1.12, 95% CI: 1.00–1.25, *P* = .045), family.*unknownfamily* (OR = 1.08, 95% CI: 1.01–1.15, *P* = .028), family.*Victivallaceae* (OR = 1.08, 95% CI: 1.01–1.15, *P* = .016), genus.*Fusicatenibacter* (OR = 1.16, 95% CI: 1.03–1.30, *P* = .011), genus.*Lachnoclostridium* (OR = 1.19, 95% CI: 1.05–1.36, *P* = .008), genus.*Prevotella9* (OR = 1.09, 95% CI: 1.00–1.19, *P* = .049), genus.*Ruminococcusgauvreauiigroup* (OR = 1.15, 95% CI: 1.03–1.29, *P* = .016), and genus.*unknowngenus* (OR = 1.12, 95% CI: 1.00–1.25, *P* = .045). Inverse MR analysis did not show significance. The *P*-values for class.*Actinobacteria*, order.*Bifidobacteriales*, family.*Bifidobacteriaceae*, and genus.*Bifidobacterium* demonstrated statistical significance. However, due to the presence of horizontal pleiotropy in order.*Bifidobacteriales* and family.*Bifidobacteriaceae*, no causal relationship could be established. Additionally, heterogeneity was observed for class.*Actinobacteria* and genus.*Bifidobacterium*, after removing outliers, the *P*-values ceased to be significant, further negating evidence of causality.

#### 3.3.3. Causal effect of gut microbiota on colorectal polyps

Based on the IVW method we identified a total of 8 gut microbiota that may be associated with colon polyps (Fig. 5). We found 1 class, 1 order, 1 family, and 5 genus with inhibitory effects on colon polypogenesis, class.*Alphaproteobacteria* (OR = 0.89, 95% CI: 0.81–0.99, *P* = .029), order.*Bifidobacteriales* (OR = 0.89, 95% CI: 0.83–0.96, *P* = .001), family.*Bifidobacteriaceae* (OR = 0.89, 95% CI: 0.83–0.95, *P* = .001), genus.*Parabacteroides* (OR = 0.84, 95% CI: 0.71–0.99, *P* = .035), genus.*Flavonifractor* (OR = 0.88, 95% CI: 0.79–0.97, *P* = .008), genus.*Adlercreutzia* (OR = 0.91, 95% CI: 0.83–1.00, *P* = .042), genus.*Victivallis* (OR = 0.93, 95% CI: 0.88–0.98, *P* = .008), genus.*Bifidobacterium* (OR = 0.93, 95% CI: 0.87–1.00, *P* = .047). Four microbiota taxa had a positive causative factor for colon polyps, order.*Pasteurellales* (OR = 1.09, 95% CI: 1.02–1.16, *P* = .015), family.*Pasteurellaceae* (OR = 1.08, 95% CI: 1.01–1.15, *P* = .026), genus.*Sellimonas* (OR = 1.09, 95% CI: 1.02–1.18, *P* = .015), genus.*Oscillospira* (OR = 1.1, 95% CI: 1.00–1.21, *P* = .049). Inverse MR analysis did not show significance.

#### 3.3.4. Causal effect of gut microbiota on gastric polyps

Based on the IVW method we identified a total of 10 gut microbiota that may inhibit the development of gastric polyps (Fig.5). Phylum.*Actinobacteria* (OR = 0.8, 95% CI: 0.63–1.00, *P* = .047), class.*Actinobacteria* (OR = 0.83, 95% CI: 0.69–0.99, *P* = .037), order.*Bifidobacteriales* (OR = 0.82, 95% CI: 0.68–0.99, *P* = .043), order.*Verrucomicrobiales* (OR = 0.79, 95% CI: 0.62–1.00, *P* = .048). family.Alcaligenaceae (OR = 0.72, 95% CI: 0.56–0.94, *P* = .014), family.Bifidobacteriaceae (OR = 0.82, 95% CI: 0.69–0.98, *P* = .033), genus.Barnesiella (OR = 0.73, 95% CI: 0.57–0.94, *P* = .014), genus.*RikenellaceaeRC9gutgroup* (OR = 0.85, 95% CI: 0.75–0.96, *P* = .012), genus.*unknowngenus* (OR = 0.74, 95% CI: 0.59–0.93, *P* = .009), genus.*Victivallis* (OR = 0.79, 95% CI: 0.69–0.91, *P* = .001). Inverse MR analysis did not show significance.

### 3.4. Sensitivity analysis

To evaluate potential horizontal pleiotropy and estimator bias, we employed MR-Egger regression and the classical IVW method of analysis. Among the 10 dietary factors exhibiting significant causality, we found no evidence of heterogeneity, and the intercept term of the MR-Egger regression did not indicate horizontal pleiotropy (Fig. 3). Additionally, we conducted a leave-one-out analysis, sequentially excluding SNPs, to assess whether the causality estimates were influenced by individual genetic instruments. The results further corroborated the robustness of the MR analysis findings in this study. Details of the leave-one-out analysis for positive findings are provided in Supplementary file S3, Supplemental Digital Content, https://links.lww.com/MD/Q445. In the analysis of gut microbiota impact on polyps, significant heterogeneity was observed in the results associated with the female genital tract polyps for the class.*Actinobacteria*, order.*Bifidobacteriales*, family.*Bifidobacteriaceae*, and genus.*Bifidobacterium*. Additionally, order.*Bifidobacteriales* and family.*Bifidobacteriaceae* exhibited concurrent horizontal pleiotropy. It is noteworthy that while the class.*Actinobacteria* was implicated as a causal factor for nasal polyps, heterogeneity in the findings was also present. Consequently, the robustness of these results is questionable.

### 3.5. Mediation analysis

**Figure 6.**
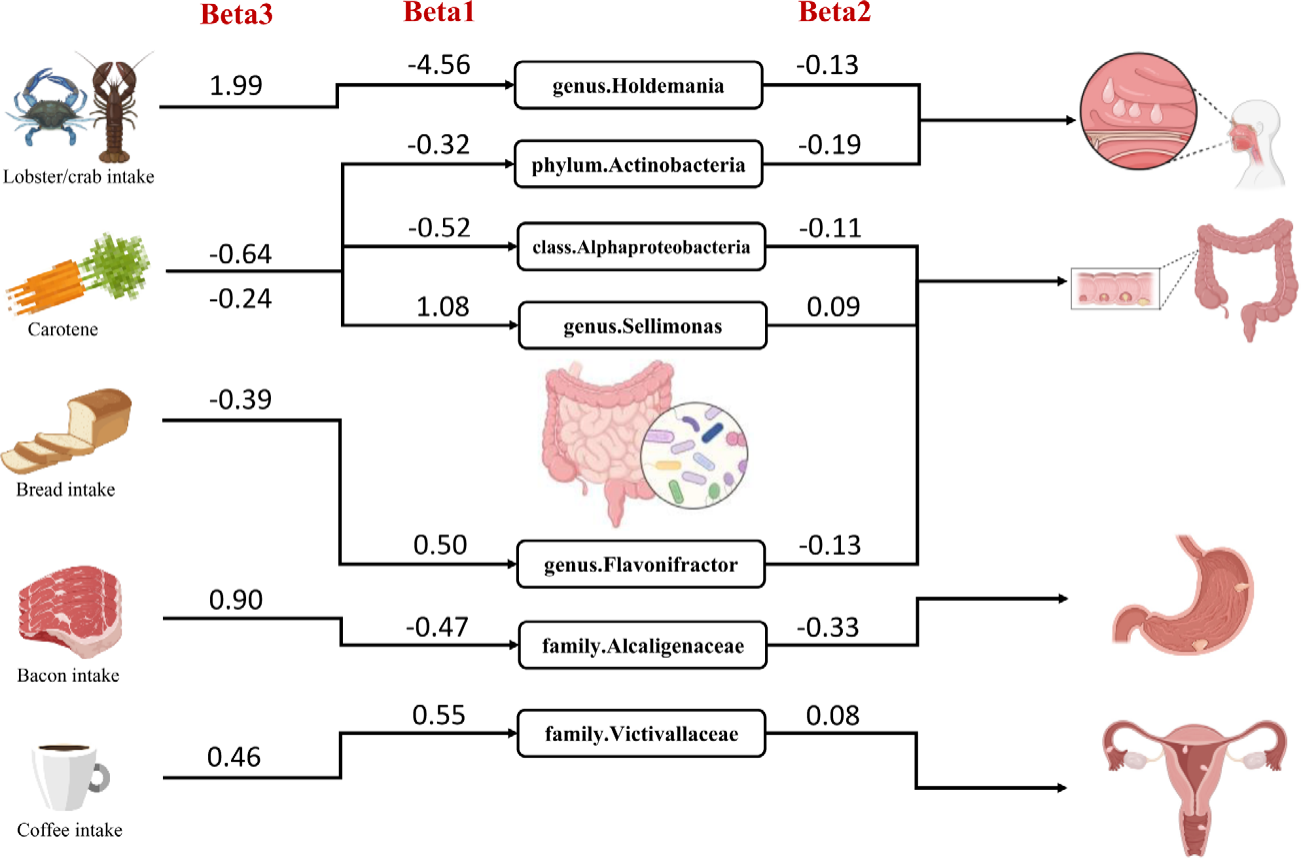
Mediating role of gut microbiota in the causal effect of dietary factors on polyps. Causal effects of dietary factors on polyps (β_3_), dietary factors on gut microbiota (β_1_), and gut microbiota on polyps (β_2_). There were a total of 4 causal pathways in which the mediating direction (β_1_*β_2_) was consistent with the direction of dietary factors-polyps (β_3_). We first identified genetic instruments for dietary factors and estimated their effects on gut microbiota (β_1_) using univariate MR. For significant microbiota, we then estimated their effects on polyps (β_2_) using independent genetic instruments. The total effect of each dietary factor on polyps (β_3_) was also estimated via univariate MR. The indirect mediated effect was calculated as β_1_β_2_, and the mediation proportion was derived as (β_1_*β_2_)/β_3_.

In univariate MR, we found a total of 10 dietary factors as well as 35 gut microbiota to have a significant causal effect on polyps. Then, we did a 2-sample MR analysis of these 10 dietary factors and 35 gut microbiota. We identified a total of 7 causal pathways in which dietary factors modulated the gut microbiota and thus influenced the development of polyps, and obtained the mediated proportion of these gut microbiota (Supplementary file S4, Supplemental Digital Content, https://links.lww.com/MD/Q445). Among them, the mediating direction of 4 causal pathways (β_1_*β_2_) was consistent with that of the dietary factor-polyp direction (β_3_; Fig. 6) An increased intake of lobster/crab may suppress the proliferation of genus.*Holdemania*, thereby potentially elevating the risk of nasal polyp development, with a mediating effect of 29.79%. The consumption of bread serves as a protective factor against the formation of colonic polyps, exerting its influence by augmenting the abundance of genus.*Flavonifractor*, with a mediating effect of 16.67%. Intake of bacon has been observed to inhibit the family.*Alcaligenaceae*, a protective factor for gastric polyps, with a mediating effect of 17.23%. Furthermore, coffee consumption is associated with an increased risk of female genital tract polyps through the enhancement of the abundance of family.*Victivallaceae*, with a mediating effect of 9.57%. The mediating effects across these 4 causal pathways are statistically significant, with some of the ratios exceeding 9%, reaching a peak of 29.79%. This significant mediation not only corroborates the key intermediary role of the gut microbiota in the influence of dietary factors on polypogenesis but also underscores the importance of the gut microbiota as a biomarker that links environmental factors to the host’s physiological responses.

## 4. Discussion

In this study, we explored the potential causal links between specific dietary patterns, diet-derived antioxidant intake, and the development of polyps in humans, including nasal, female genital tract, colonic, and gastric polyps. We also investigated the mediating role of gut microbiota in this relationship. Through univariate MR analysis, we identified 7 dietary patterns – lobster/crab, bacon, processed meat, cheese, coffee, bread, and beef intake – and 3 diet-derived antioxidant sources – vitamin B_6_, vitamin C, and carotene – that were significantly and causally linked to polyp development. Furthermore, this study uncovered a correlation between 35 types of intestinal microbiota and polypogenesis. Although a significant *P*-value was observed, substantial heterogeneity and horizontal pleiotropy preclude a reliable causal interpretation for the role of class.*Actinobacteria* in female genital tract, nasal, and gastric polyps. Notably, the family.*Bifidobacteriaceae* and order.*Bifidobacteriales* showed potential inhibitory effects on gastric and colon polyps. Although preliminary results suggest that family.*Bifidobacteriaceae*, order.*Bifidobacteriales* may promote female genital tract polyps, the robustness of this finding is equally challenged by the presence of horizontal pleiotropy and heterogeneity. The genus.*Bifidobacterium* demonstrated inhibitory effects on nasal and colon polyps. The class.*Alphaproteobacteria* exerted varying causal effects, inhibiting colon polyps while positively influencing female genital tract polyps. This is a fascinating discovery. In IBD patients, the abundance of phylum.*Proteobacteria* is significantly higher than in healthy individuals.^[39]^ Research also shows that a high-salt diet increases the abundance of class.*Alphaproteobacteria* in the gut. Previous reports indicate that after FMT treatment, the abundance of class.*Alphaproteobacteria* rises significantly, suggesting it may have a protective function.^[40]^ However, multiple MR studies^[41,42]^ have pointed out that this class of bacteria may increase the risk of postpartum depression and renal cell carcinoma. This study suggests that class.*Alphaproteobacteria* may have a protective effect on colon polyps but increase the risk of genital tract polyps. This organ-specific effect may arise from interactions between this bacterial group and sex hormone metabolism, thereby specifically altering the local environment of the genital tract. The genus.*RikenellaceaeRC9gutgroup* and phylum.*Actinobacteria* both inhibited the development of nasal and gastric polyps. Additionally, genus.*Victivallis* inhibited the occurrence of colon polyps as well as gastric polyps. Moreover, the study identified 4 causal pathways linking diet, gut microbiota, and polyps. These findings shed new light on the intricate interplay among dietary factors, gut microbiota, and polyps, suggesting potential intervention targets for future research and clinical applications.

Dietary components undergo transformation and absorption in the digestive tract, affecting the metabolic state of the human body through direct and indirect effects. These effects include influencing the contact and transport time between food components and the mucosal lining of the digestive tract, as well as promoting the production of SCFAs, among others. The large cohort study of the European Prospective Investigation into Cancer and Nutrition (EPIC-Oxford) found that the incidence of colorectal cancer was higher among vegetarians compared to meat-eaters.^[43]^ The results of the study showed that bread intake inhibited the formation of colorectal polyps. This finding aligns with previous studies,^[44]^ which concluded that components generated during bread fermentation act as in vitro prebiotics and contribute to maintaining intestinal stability. Another study^[45]^ revealed that consuming sustainably fermented yeast bread also promotes the metabolism of a healthy colonic microbiota. Carotene is important diet-derived antioxidants that are widely present in vegetables and fruits consumed by humans. Numerous studies have demonstrated that carotene improves cognitive function, cardiovascular health, and eye health, while exhibiting potential for the prevention and treatment of many chronic diseases. A clinical study^[46]^ revealed that carotenoid levels in the intestinal tissue of colon cancer patients were lower compared to healthy controls and patients with colon polyps. In vitro experimental evidence^[47]^ suggests that β-carotene may exhibit anti-colon cancer effects by regulating M2-type macrophages and activating fibroblasts. Animal studies have also demonstrated^[48]^ that β-carotene can reduce the phosphorylation levels of pro-inflammatory cytokines (e.g., p65, p38, Erk, and JNK), thereby regulating the gut microbiota and exerting anti-inflammatory effects. Consistent with the above findings, the results of the univariate MR analysis in the present study also suggested that carotenoid intake was beneficial to the intestinal tract and reduced the formation of colorectal polyps.

Similar to the intestinal tract, the gastric mucosa is in direct contact with food, and food components may directly influence the barrier function and repair ability of the gastric mucosa. Previous studies^[49,50]^ revealed that ascorbic acid and total blood vitamin C concentrations in gastric fluid were lower in patients with gastritis than in healthy people, and that was more significant in patients infected with *Helicobacter pylori*. A meta-analysis^[51]^ demonstrated that vitamin C taken with vitamin E reduced the incidence of gastric polyps by 7%. By following 3365 residents from high-risk areas for gastric cancer over 22 years, Li et al^[52]^ found that the incidence of new gastric cancer cases and gastric cancer-related deaths was significantly reduced with vitamin C supplementation compared to other patients. Our study further demonstrates the important link between vitamin C and gastric polyp, and suggests that dietary patterns may influence the occurrence of gastric polyps by modulating genetic susceptibility. Additionally, beef intake exhibited significant associations in both LDSC and univariate MR analyses. A meta-analysis of 43 studies by Kim et al^[53]^ concluded that the risk of gastric cancer increased by 1.26 for every 100 g/d increase in red meat intake and by 1.72 for every 50 g/d increase in processed meat intake, suggesting that high consumption of red or processed meat may increase the risk of gastric cancer. A diet high in meat may also be a potential risk factor for gastric polyps.^[54]^ Additionally, a large cross-sectional study^[55]^ found that red meat intake is a risk factor for colorectal polyps, particularly serrated polyps.

Coffee is one of the most widely consumed beverages globally, and its association with various health outcomes has garnered significant attention. A large meta-analysis^[56]^ demonstrated that coffee consumption within the typical range of intake is safe, with 3 to 4 cups of coffee per day being associated with the greatest health benefits. Another MR study^[57]^ based on genetic associations found no strong evidence supporting a causal link between genetically predicted coffee consumption levels and most of the studied cancers. A 16-year study by McCann et al^[58]^ involving 1082 women demonstrated that consuming more than 4 cups of coffee and tea per day was significantly associated with a reduced risk of endometrial cancer. Cheese is an important source of high-quality protein, calcium, vitamin B_12_ and other nutrients that are essential for women’s health at all life stages. Moderate intake of cheese is beneficial for bone development, prevention of osteoporosis, promotion of muscle synthesis and maintenance of muscle mass, and contributes to energy metabolism balance.^[59,60]^ However, cheese is also a high-fat, high-calorie food, and excessive intake may increase the risk of obesity and related metabolic diseases, which may adversely affect women’s physical health and fertility.^[61]^ In a large cohort study^[62]^ of 52,795 individuals, higher dairy calorie and milk intake led to an increased incidence of breast cancer. A more nuanced conclusion was reached in a study by Wajszczyk et al,^[63]^ where a statistically significant, but directionally different, association was found between the same dairy products and breast cancer risk, with differences in pre and postmenopausal women. In the absence of clinical studies on the association between coffee, cheese and female genital tract polyps, we highlight this diet-gene interaction suggesting that excessive coffee and cheese intake increases the risk of female genital tract polyps and that individualized dietary patterns may be better able to prevent the development of the disease.

Notably, the effect size of lobster/crab intake on the risk of nasal polyps was 1.99, indicating a moderate effect magnitude. Meanwhile, the mediation effect proportion of Holdemania in this pathway reached 29.79%, a substantial magnitude, suggesting that the mechanism by which lobster/crab influences the occurrence of nasal polyps largely depends on Holdemania. Holdemania is recognized as a normal member of the human gut microbiota and plays a critical role in tryptophan metabolism.^[64]^ Studies^[65,66]^ have found that the abundance of Holdemania in the gut of patients with cognitive dysfunction is significantly reduced, while this microbiota is significantly enriched in patients responsive to tumor immunotherapy. Importantly, the abundance of Holdemania is susceptible to modulation by dietary components, particularly excessive intake of sucrose and fructose, which can lead to a significant increase in its abundance.^[67]^ The effect size of coffee intake on the risk of reproductive tract polyps was 0.46, also indicating a moderate effect magnitude. Although the mediation effect of Victivallaceae was only 9.57%, the OR was 1.15 with a 95% CI of 1.15 to 2.18, indicating a highly stable effect size. Given the large global population of female coffee drinkers, even with a relatively small absolute value of this mediation effect, it may still exert practical impacts on a substantial population through the gut microbiota pathway.

Diet is not only a key factor in shaping the structure of the gut microbiota, but also has a significant impact on host health through the metabolism of gut microbes, which produce derivatives or metabolites.^[68]^ The human gut microbiota is intimately connected to dietary habits, with its composition varying notably with the intake of fats and fibers.^[69]^ Elevated intake of saturated fats and sucrose, coupled with low fiber consumption, can alter the structure of microbial taxa and raise the incidence of metabolic disorders such as diabetes and obesity, particularly in Westernized societies.^[70,71]^ Olson et al^[72]^ suggested that ketogenic diets might exert their therapeutic effects on refractory epilepsy through modulating the levels of *Akkermansia* and *Parabacteriodes* in the gut microbiota. Furthermore, another research^[73]^ indicates that the Mediterranean diet can reduce *Prevotella* levels while enhancing *Roseburia* and *Oscillospira*, thereby improving insulin sensitivity in obese individuals. This discovery underscores the gut microbiota’s regulatory influence on the host’s metabolic processing and response to dietary components, elucidating how dietary elements, by shaping gut microbial composition and function, reciprocally impact the host’s physiological and pathological conditions. The significant mediating effects imply that the gut microbiota could serve as a sensitive indicator of dietary impacts on polyp development, with its alterations potentially signaling early shifts in the influence of dietary patterns on human health. Moreover, this outcome offers a scientific rationale for formulating nutritional interventions aimed at the gut microbiota, potentially preventing or mitigating polyp progression and consequently lowering the risk of precancerous conditions.

Our study has several limitations that should be considered. First, our analysis was primarily based on GWAS summary data from participants of European ancestry. Consequently, the generalizability of our findings to non-European populations remains uncertain and warrants future investigation in more diverse ethnic groups. Second, although the MR design reduces susceptibility to conventional confounding and reverse causation, it relies on the critical assumption of no unmeasured confounding. Despite employing robust sensitivity analyses to mitigate pleiotropic bias, we cannot completely rule out the potential for residual confounding by unmeasured factors.

## 5. Conclusion

Our study provides evidence suggestive of potential causal relationships between specific dietary factors and polyps. Most importantly, our MR analysis supports the hypothesis that 7 dietary patterns and 3 diet-derived antioxidants may influence polyp development, potentially mediated through 35 gut microbiota taxa. Our findings highlight the potential for polyp prevention through rational dietary modifications and warrant further investigation to confirm these associations.

## Acknowledgment

The author would like to thank Haijuan Xiao for reviewing this article and providing detailed suggestions.

## Author contributions

**Data curation:** Ying Zou.

**Funding acquisition:** Guankai Lin.

**Methodology:** Cheng Zhou.

**Project administration:** Shenkang Tang.

**Supervision:** Kaiting Sun.

**Validation:** Shuhan Yang.

## Supplementary Material


